# Quantifying Bone and Skin Movement in the Residual Limb-Socket Interface of Individuals With Transtibial Limb Loss Using Dynamic Stereo X-Ray: Protocol for a Lower Limb Loss Cadaver and Clinical Study

**DOI:** 10.2196/57329

**Published:** 2024-04-26

**Authors:** Jason T Maikos, John M Chomack, David V Herlihy, David N Paglia, Charlene Wetterstrand, J Patrick O'Connor, Michael J Hyre, J Peter Loan, Susan E D'Andrea

**Affiliations:** 1 Veterans Affairs New York Harbor Healthcare System New York, NY United States; 2 Narrows Institute for Biomedical Research and Education, Inc. Brooklyn, NY United States; 3 Department of Orthopaedics Rutgers-New Jersey Medical School Newark, NJ United States; 4 C-Motion, Inc Germantown, MD United States; 5 Department of Kinesiology College of Health Sciences University of Rhode Island Kingston, RI United States

**Keywords:** biplanar fluoroscopy, dynamic stereo x-ray, lower limb loss, transtibial limb loss, prosthetic sockets, amputation, lower extremity

## Abstract

**Background:**

Relative motion between the residual limb and socket in individuals with transtibial limb loss can lead to substantial consequences that limit mobility. Although assessments of the relative motion between the residual limb and socket have been performed, there remains a substantial gap in understanding the complex mechanics of the residual limb-socket interface during dynamic activities that limits the ability to improve socket design. However, dynamic stereo x-ray (DSX) is an advanced imaging technology that can quantify 3D bone movement and skin deformation inside a socket during dynamic activities.

**Objective:**

This study aims to develop analytical tools using DSX to quantify the dynamic, in vivo kinematics between the residual limb and socket and the mechanism of residual tissue deformation.

**Methods:**

A lower limb cadaver study will first be performed to optimize the placement of an array of radiopaque beads and markers on the socket, liner, and skin to simultaneously assess dynamic tibial movement and residual tissue and liner deformation. Five cadaver limbs will be used in an iterative process to develop an optimal marker setup. Stance phase gait will be simulated during each session to induce bone movement and skin and liner deformation. The number, shape, size, and placement of each marker will be evaluated after each session to refine the marker set. Once an optimal marker setup is identified, 21 participants with transtibial limb loss will be fitted with a socket capable of being suspended via both elevated vacuum and traditional suction. Participants will undergo a 4-week acclimation period and then be tested in the DSX system to track tibial, skin, and liner motion under both suspension techniques during 3 activities: treadmill walking at a self-selected speed, at a walking speed 10% faster, and during a step-down movement. The performance of the 2 suspension techniques will be evaluated by quantifying the 3D bone movement of the residual tibia with respect to the socket and quantifying liner and skin deformation at the socket-residuum interface.

**Results:**

This study was funded in October 2021. Cadaver testing began in January 2023. Enrollment began in February 2024. Data collection is expected to conclude in December 2025. The initial dissemination of results is expected in November 2026.

**Conclusions:**

The successful completion of this study will help develop analytical methods for the accurate assessment of residual limb-socket motion. The results will significantly advance the understanding of the complex biomechanical interactions between the residual limb and the socket, which can aid in evidence-based clinical practice and socket prescription guidelines. This critical foundational information can aid in the development of future socket technology that has the potential to reduce secondary comorbidities that result from complications of poor prosthesis load transmission.

**International Registered Report Identifier (IRRID):**

DERR1-10.2196/57329

## Introduction

### Background

For individuals with lower limb loss who use a prosthesis, relative motion between the prosthetic socket and the residual limb, including vertical translation and axial rotation, is a common problem that can lead to skin integrity concerns [1]. Excessive motion between the residual limb and socket can lead to discomfort, pain, and gait deviations that can limit mobility [2], which has been correlated with worse quality of life [3]. Up to 40% of individuals with transtibial limb loss experience issues involving the residual skin and soft tissue, which can be directly attributed to the movement of the residuum relative to the prosthetic socket and liner during dynamic activities [4]. Restricting residual bone motion within the socket may therefore help improve the quality of life and comfort for individuals with lower limb loss. Although some efforts have been made to advance socket technology [5-7] and improve suspension techniques [8], clinical practice has been slow to adopt these new technologies and continues to primarily rely upon unscientific methods for socket fabrication [9,10]. The challenge of developing enhanced socket technology and suspension techniques can be partially attributed to the multifactorial nature of socket fit, which is complicated by the complex mechanical interaction between the residual limb (bone and tissue), the liner, and the socket during dynamic activities. Furthermore, there has traditionally been a lack of accurate analytical techniques to quantify the complex, dynamic biomechanics of the socket-residual limb interface.

Although biomechanical assessments of the relative motion between the residual limb and the prosthetic socket have been performed, the existing data are suboptimal or lack an appropriate resolution. Radiological methods have been widely used to measure the relative motion of the bone and socket in 2 dimensions, but these methods have only provided static analyses of the residual limb-socket relationships [11,12]. Ultrasound has been used to monitor residual limb motion in individuals with above-knee limb loss [13], but the mounting technique has been found to be too cumbersome to implement on a clinical basis. Advanced imaging methods including computed tomography (CT) and spiral x-ray CT offer higher spatial resolution, but imaging must be performed in the supine position, resulting in non–weight-bearing, static assessments [14-16]. Roentgen stereophotogrammetric analyses have been used to characterize the motion between the residual limb and socket in 6 degrees of freedom (DOF) by using biplanar imaging [17]; however, these techniques use static loading protocols. Dynamic assessments of 3D in-socket residual limb-socket kinematics are currently only possible using dynamic stereo x-ray (DSX), which can provide submillimeter bone pose [18-21] and in vivo strain analysis. Currently, only 1 study performed a dynamic investigation of residual tibia motion in participants with transtibial limb loss [10], but the methods relied on subjective input and were time intensive, which can affect accuracy [22] and sample size. In individuals with transfemoral limb loss, Maikos et al [23] used DSX to compare 3D residual femur kinematics between 2 different prosthetic socket types. Gale et al [3] also used DSX for 3D markerless tracking of the residual femur for individuals with transfemoral limb loss during late swing and early stance to calculate the 6 DOF kinematics of the residual femur relative to the socket but did not include terminal stance. DSX may help fill the substantial gap in our understanding of the complex mechanics of the residual limb-socket interaction during dynamic activities that limit the ability to improve prosthetic design.

During the cyclical loading and unloading of the residual limb during the gait cycle, the skin of the residual limb is exposed to nonphysiological stresses and strains, including excessive shear forces [24]. Although the skin is well adapted to compressive force, excessive shear force can be damaging, leading to abrasions, wounds, and ulcers [25,26]. Understanding in-socket skin strain biomechanics is critical for enhancing prosthetic socket fit, limb health, and overall comfort. However, whole-limb skin strain analysis is complicated by the heterogeneous composition of the skin and its anisotropic mechanical properties. Some investigations have used 3D digital image correlation (DIC) to create full-field deformation and strain maps of an unsupported residual limb [27]. Lin et al [28] examined skin strain in a flexed biological residual limb from an individual with transtibial limb loss and showed that the anterior patella region of the limbs exhibited predominantly tensile strains, whereas the posterior patella region exhibited predominately compressive strains. Although 3D DIC evaluations have provided critical data to help improve the mechanical interface of sockets and liners to limit relative motion and shear forces on the skin surface, these investigations only considered strain on an unloaded residual limb. Whole-limb strain fields can drastically change during dynamic, weight-bearing activities while using a prosthetic interface. Furthermore, 3D DIC and other imaging techniques are further complicated by the need for transparent biomechanical interfaces to accurately compute the strain analysis. Other techniques, such as magnetic resonance imaging and CT, can be used to evaluate in vivo strains, but they are also limited by static protocols, low resolution, motion artifacts, and shape distortion [29]. Gale et al [30] used DSX to measure residual limb skin strain and strain rate for individuals with transfemoral limb loss during gait and found that shear strain increased from proximal to distal regions of the residual limb. The proposed investigation will use time-efficient and highly accurate analytical techniques to measure in-socket residual limb skin strain for individuals with transtibial limb loss during dynamic activities.

### Study Objectives and Aims

There remains an unmet need to fill the gap of accurate, biomechanical evaluations of residual limb-socket kinematics, which can then be effectively translated into evidence-based clinical practice. Furthermore, quantifying dynamic shear and tissue deformation is yet to be efficiently evaluated to determine the exact mechanism of tissue strain. Therefore, the objective of this investigation is to develop quantitative, dynamic analytical tools to quantify both 3D bone movement as well as soft tissue and liner deformation at the socket-residual limb interface for individuals with transtibial limb loss. To meet the study objective, the following aims will be addressed: (1) to optimize the DSX procedural setup for the accurate tracking of the prosthetic socket, skeletal kinematics, and tissue and liner deformation; (2) to quantify the relative motion between the residual tibia and the prosthetic socket during dynamic activities; and (3) to measure the deformation of the skin and liner in the prosthetic socket during dynamic activities. The proposed investigation will use a state-of-the-art DSX system to accurately quantify 3D in vivo residual limb-socket kinematics during dynamic activities. To verify the sensitivity of this technique and its relevance to individuals with transtibial limb loss, residual limb-socket kinematics will be evaluated in 2 different socket suspension systems: elevated vacuum (EV) and traditional suction. It is hypothesized that an efficient and highly accurate method to quantify the dynamic interaction between the residual limb and the prosthetic socket will be sensitive enough to distinguish between different types of prosthetic socket suspension systems, which will further enhance the biomechanical understanding of residual limb-socket kinematics.

## Methods

### Study Overview

To address the study aims, first an iterative cadaver study will be conducted to optimize the placement of an array of radiopaque beads and markers on the socket, liner, and skin to simultaneously assess both dynamic skeletal movement and residual skin and liner deformation. Using a gait simulator, stance phase gait will be simulated using cadaver limbs during each DSX session to induce bone movement as well as skin and liner deformation. The number and placement of markers will be evaluated after each session to refine the marker placement to best track skin and liner deformation and skeletal movement. Once an optimal marker setup is identified, 21 participants with transtibial limb loss will be fitted with a socket capable of being suspended via both EV and traditional suction. Participants will undergo a 4-week acclimation period using the new socket and will then be evaluated at the DSX facility at Rutgers New Jersey Medical School. DSX will be used to track skeletal, skin, and liner motion under both suspension techniques during 3 dynamic activities: treadmill walking at a self-selected speed, treadmill walking at fast walking (10% faster), and a step-down movement. The performance of the 2 suspension techniques (active EV and traditional suction) will be tested by quantifying the 3D bone movement of the residual tibia with respect to the prosthetic socket and quantifying liner and skin deformation at the socket-residuum interface. This study has been registered on ClinicalTrials.gov (NCT05287646).

### Cadaver Study to Optimize DSX Setup

The aim of the cadaver study is to optimize the placement of an array of radiopaque beads and markers on the socket, liner, and skin, which will then be used in the human trial to accurately measure the dynamic skeletal movement and residual tissue and liner deformation simultaneously during functional tasks. Five cadaver limbs will be used in an iterative process to develop an optimal marker setup to distinguish markers placed on the socket, liner, and skin as well as to visualize the tibia. The number, size, shape, and placement of markers will be evaluated after each cadaver test to determine the optimal marker set for the measurement of skin and liner deformation and bone movement. The goal is to develop a skin and liner marker set that is free of occlusion during tracking while also not interfering with bone tracking. Although it is understood that the cadaveric tissue may not produce accurate tissue displacement and deformation profiles compared to living tissue, the main intent of cadaver testing is to accurately position the skin and liner markers during an iterative process to reduce or eliminate occlusion during tracking.

### Cadaver Preparation and Socket Casting

Five fresh-frozen, whole, lower extremity cadaveric specimens without a history of significant trauma or major surgery at or below the knee will be used. Lower extremity cadaver specimens will be from individuals aged <80 years and with a BMI of <38 kg/m^2^ to account for the predicted upper limits of the human trials. Cadavers will be thawed at room temperature for 24 hours and will be transected at the midthigh to keep the knee intact. The cadaver specimens for the initial assessments will be amputated below the knee to a length between 12.5 cm and 17.5 cm below the medial joint line, which is considered an ideal length for transtibial amputation [31]. Myodesis will be performed to stabilize the muscles, and the remaining skin flap will be sutured in place. After initial cadavers of ideal length have been used to determine an optimized marker set, subsequent cadaver limbs will be amputated at shorter lengths to ensure that the marker set does not need to be modified to account for different residual limb lengths. A liner will be placed over the amputated cadaver limbs and then cast with fiberglass to create a negative mold. The negative mold will be filled with plaster to create a positive cast, which will be used to fabricate a ThermoLyn socket. Cadavers will be fitted with the socket and prosthetic componentry, including a pylon and prosthetic foot. A rocker bottom shoe will be placed on the prosthetic foot to aid in simulating dynamic gait.

### Cadaver CT Scans

A single CT scan will be acquired for each cadaver experiment. The residual limb will be marked with radiopaque paint. A silicone liner will be applied to the residual limb, and a thermoformed plastic socket marked with solid, 2-mm diameter embedded brass spheres will be placed on the limb before scanning. The socket will maintain the unloaded 3D shape of the residual limb and the relative positioning of the radiopaque makers. The CT scan will be used to create gray-scale volumes and high-resolution 3D bone surface models required for computing all outcome variables in DSX. CT volume images will be acquired at a resolution of 512×512×0.625 mm^3^ (120 kVp; SMART mA).

### Experimental Design

To determine the areas of highest strain on both the skin and inner liner surface (to serve as a starting point for appropriate marker placement), the first cadaver experiment will use a grid of circular radiopaque markers distributed across the circumference of the residual limb and the inner surface of the liner (in separate experiments). Although this grid pattern will cover the entire surface to help quantify areas of highest strain on the liner and skin, the high density of radiopaque markers will make it unlikely to track the movement of the residual tibia, liner, skin, and socket accurately and simultaneously. Therefore, this initial cadaver experiment will serve only to help determine areas of importance for subsequent cadaver studies. However, previously reported skin strain findings from the scientific literature will be considered to ensure that areas of interest are included [10,27]. For clarity, the procedures will be first carried out with markers on the skin and no markers on the liner. The experiment will then be repeated using a liner with no markers on the skin.

A custom grid stencil will be fabricated by cutting specific patterns of circles on a thin vinyl sheet using an electronic die-cutting machine (Cricut, Inc). The circles on the grid will be cut to diameters of either 2 mm or 4 mm and spaced 2 cm apart within each row and column. Each row and column of circles will alternate between the larger and smaller circles, which will help uniquely identify them during marker tracking. The stencil will then be applied to the cadaver limb or liner (in separate experiments), and radiopaque paint will be applied to the pattern to transfer the grid onto the residual limb or liner. The liner will then be placed over the limb, the socket will be donned, and the limb will be placed in the DSX capture volume. Averaging the DSX data collected from 10 to 20 frames from the unloaded-donned socket in the static pose will produce the relative resting grid positions, which will account for any skewness after donning the liner and socket. The circles will then be digitized in dynamic trials to relate their dynamic positions to the resting grid.

### Stance Simulation

**Figure 1:**
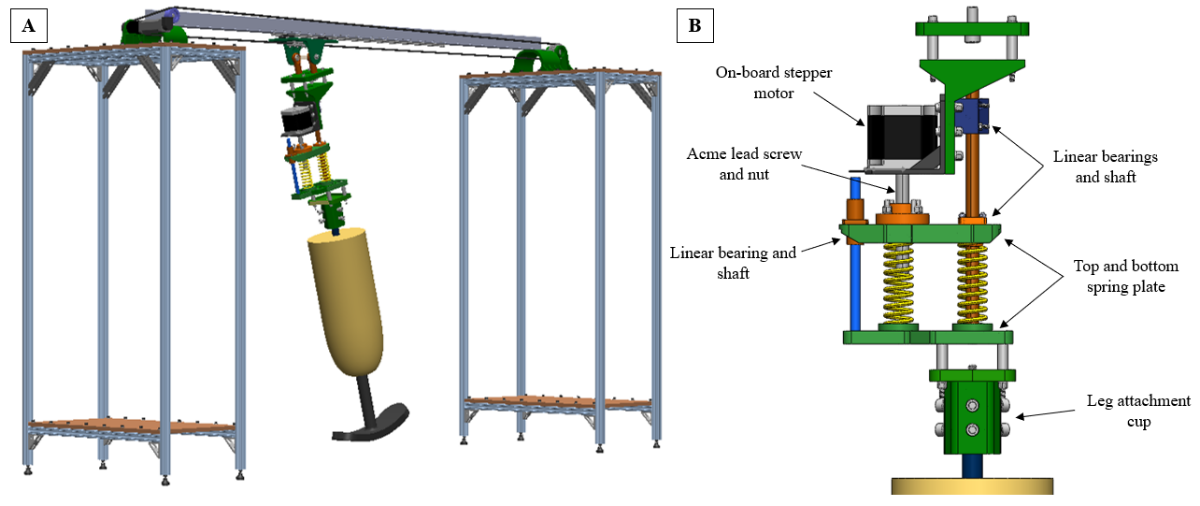
Overview of the stance simulator design for simulation of the stance phase for cadaver testing. (A) Isometric view of the stance simulator assembly. (B) Detailed view of the force applicator mechanism.

An electromechanical stance simulator will be designed to apply a compressive load on a cadaver limb through simulated stance phase (Figure 1). The framework will be designed to interface with the DSX system while allowing for remote control of the load and trajectory of a cadaver limb during DSX data capture. Using this setup in conjunction with DSX data capture, simulated stance phase will be performed to determine areas of highest strain on both the skin of a cadaver leg and the inner liner surface, while simultaneously tracking tibial movement during dynamic activities during the clinical trial. To ensure accurate results, the system will be able to apply 100% body weight in compression to induce tissue deformation. The applied load will be monitored using a force plate (AMTI Inc).

To avoid interference with the DSX system, the main support structure of the simulator will consist of 2 stands with an angle bar spanning the length between them (Figure 1A). Weights placed on the outer stands will ensure that the system remains fixed despite varied applied loads. A linear track attached to the beam will provide a low friction path for the leg to travel along the plane of forward progression, and the beam will ensure minimal deflection of the track. The leg will pivot around this attachment point, allowing it to attain a full range of motion to mimic heel-strike to toe-off. The drive motor will be placed off-board and will move the leg with a timing belt and pulley. A second, on-board motor will control the deflection of 2 springs to actuate the load applied to the leg. The force applicator mechanism (Figure 1B) will include a stepper motor with a built-in Acme lead screw and nut to add spring compression, allowing for consistent control of applied forces at high loads. In addition, 3 linear bearings in the assembly allow the assembly to compress without direct control from the on-board motor because of the natural compression of the springs as the leg rolls over in stance under the fixed-height track. The cadaver leg will be placed in extension to limit bending of the knee or the knee will be fixed in place with a titanium rod, increasing the lever arm and ensuring that the springs apply a compressive force. A 3D-printed cup fixture will be secured to the exposed femur of the leg by tightening 6 separate bolts. This attachment method will allow for alignment flexibility, varying the load path through the leg. The entire system will be controlled electronically by 2 separate motor drivers, 1 each for the off-board motor and on-board stepper motor.

### Marker Tracking

For each cadaver trial, the images will be analyzed using Locate3D (C-Motion, Inc), which is an application in the DSX suite of software. Locate3D tracks radiopaque markers in x-ray trials by locating the weighted center of circular regions on the images. The circles will be digitized in both the unloaded (resting) and loaded dynamic conditions to relate dynamic positions to the resting grid. Each circle will be tracked throughout the simulated stance phase to calculate the 3D position of each circle in the socket reference frame during dynamic movement. Point clouds will then be calculated in a common frame. Vector fields (ie, movement of the circles) will be computed between the loaded and the resting trial. The distance between each circle and its 8 closest neighbors will be calculated and measured from the resting to the loaded conditions during dynamic trials. From these measurements, the circle clusters that have the largest changes in distance will be determined. The displacement field on the limb surface will be calculated by correlating the time series of images during the dynamic movements. From the correlated skin displacement field, the Green-Lagrange strain and Euler-Almansi strain will be calculated. The areas of the highest strain from these trials will provide a strong estimate of the most appropriate placement of markers on the skin and liner for the human trials.

### Subsequent Cadaver Experiments

**Figure 2:**
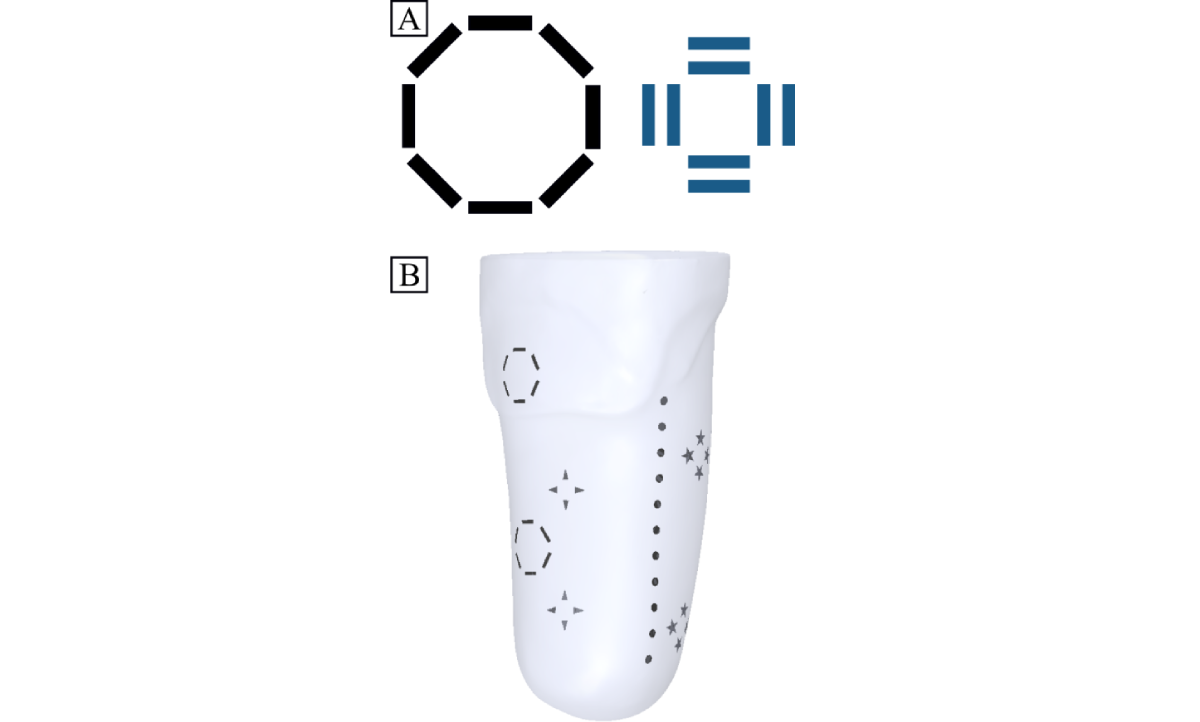
Example skin marker shape and setup for the cadaver trials. (A) Circles (lines: 10 mm in length, 3 mm wide) and squares (lines: 10 mm in length, 3 mm wide) will be placed on the skin and liner, respectively. (B) Example marker placement on the skin on a scanned, translucent residual limb in the anterior-lateral view.

**Figure 3:**
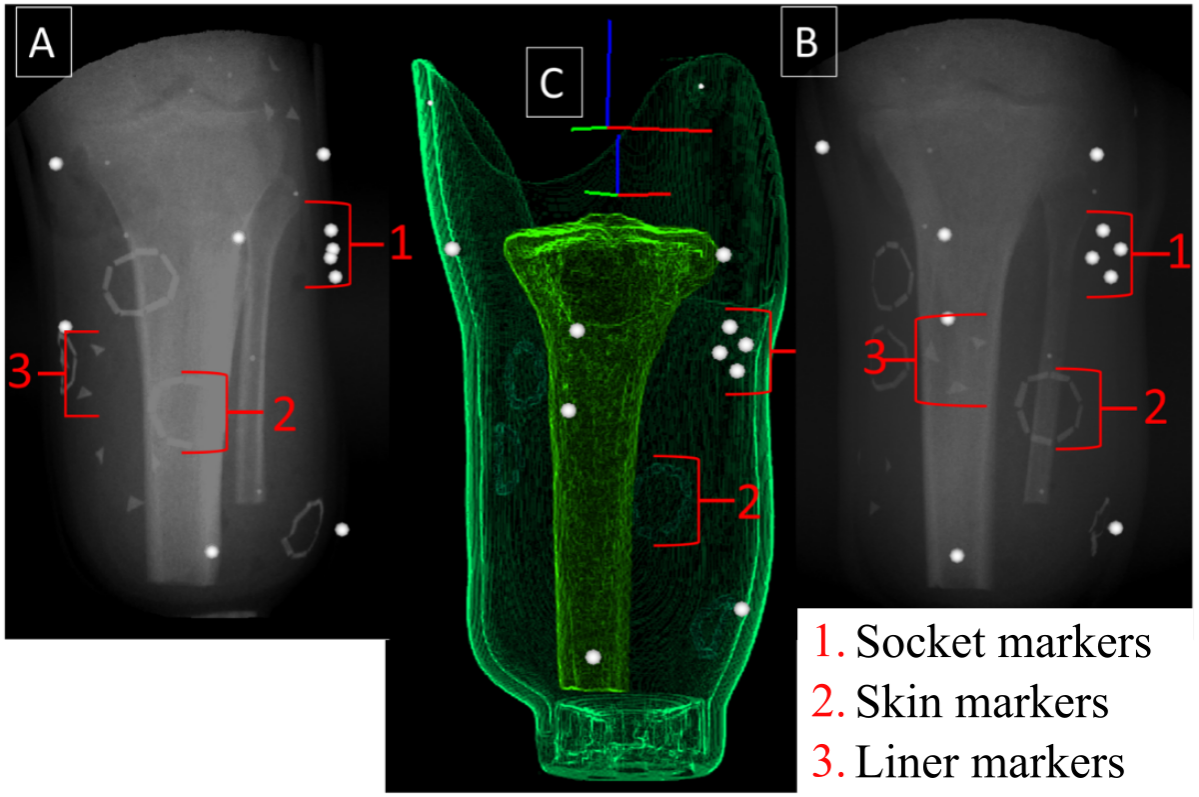
Static testing of a sample cadaver marker set in the dynamic stereo x-ray system. (A) and (B) X-ray images of the residual tibia, socket, and skin markers from the inline and offset x-ray views. Note the cluster of skin (lines in the shape of a circle), liner (triangles), and socket (beads) markers and the bone edges. (C) The x-ray and computed tomography images are fused to create a 3D model with coregistration of the socket and skin markers and bone geometry.

The results from the initial cadaver experiment will help inform the placement of markers on the liner and skin for subsequent cadaver experiments. Figure 2 illustrates example marker shapes (Figure 2A) and the setup on a cadaver limb (Figure 2B). To track the pose of the socket, brass beads (2 mm diameter) will be secured to the socket. The initial marker shapes on the skin will be lines (10×3 mm) of radiopaque paint that will be placed in a circular pattern (6 cm in diameter—black lines in Figure 2A). Lines organized in the shape of a circle were chosen because the end points of each line in each marker cluster will be able to be independently tracked. However, there is a possibility that there may be a substantial overlap between markers on the surface of the skin, particularly on opposite sides, which would limit their ability to be tracked throughout the movement trials. Therefore, additional shapes (eg, triangles and stars) will also be tested during initial cadaver experiments to determine which marker types are easiest to track throughout the movement trials. Overall, these sizes and shapes were chosen to provide a large surface area on the skin while also permitting visualization of the tibial bone edges. For the inner surface of the liner, radiopaque paint consisting of double lines (10×3 mm) in the shape of a square will be used (Figure 2A). The number of markers, marker shape, placement, and size will be evaluated after each testing session to determine the optimal placement to avoid any overlapping or occlusion of the bone edges. If the markers overlap or interfere with DSX, the sizes will be reduced, the placement of the markers will be changed, or different shapes will be used. An initial, sample cadaver marker set was tested in the DSX system under static conditions to determine if the DSX software had the ability to distinguish markers placed on the socket, skin, and liner (Figure 3). Brass beads were embedded in the socket and radiopaque markers were placed on the skin and liner (similar to that presented in Figure 2). The anteroposterior and oblique x-ray views were fused with the CT imaging into a 3D entity with coregistration of the socket markers, liner markers, skin markers, and bone geometry. It was confirmed that the DSX software could distinguish markers on all surfaces. Subsequent cadaver testing will be performed under dynamic conditions while the skin, liner, and socket markers are tracked in conjunction with bone tracking throughout the simulated stance phase.

### Optimization of the Marker Set

An iterative, heuristic approach will be used with the goal of accurately tracking all end points of each line for the skin and liner marker sets without (or minimal) occlusion while simultaneously tracking the underlying tibial movement. Notably, if there is too much overlap of the lines and tracking is not possible with the lines, additional shapes will be evaluated, and the centroid of each shape will be tracked. Cadaver trials will be sequential, and the marker set will be evaluated through tracking of each marker and performing preliminary deformation analysis after each cadaver trial. Once an acceptable marker protocol has been achieved heuristically, it will be tested on subsequent cadavers to ensure that it is robust for differing anatomies and residual limb lengths. Shorter residual limbs could potentially limit the number of markers that could be applied to the skin. As such, a minimum length may be required for skin and liner marker tracking, which would be then reflected in the inclusion and exclusion criteria.

### Deformation Analysis

The markers on the cadaver skin and the inner surface of the liner will be tracked during the simulated stance phase, and their trajectories in both the socket and tibia coordinate systems will define the motion of the skin relative to the socket and tibia, respectively. Their positions in the socket and tibia coordinate systems during the static trial will serve as their baseline positions. Visual3D (C-Motion, Inc) contains the tools to define the socket and tibia coordinate systems and track the motion of the skin and liner markers within these reference frames. Two metrics will be used to quantify skin and liner deformation. The first, shear, is defined as the change in angle between the lines painted on the skin and liner from the baseline position. The end points of the lines forming the circles and squares will be digitized, and the 3D trajectories will be used to reconstruct the 3D shape of the line at each time frame. The angles between the lines will be measured in the static pose to quantify the baseline skin shear. The change in angles will be measured during the simulated stance phase. Shear will be measured as the change in angle relative to the angle in the static position. The second, compression, will be quantified by calculating the change in distance between the lines and a neutral position. As with shear, baseline distances will be calculated from the static trial. From these values, 3D strain maps of the highest areas of deformation will be developed for both the liner and the skin.

### Validation of DSX Strain Measurements

**Figure 4:**
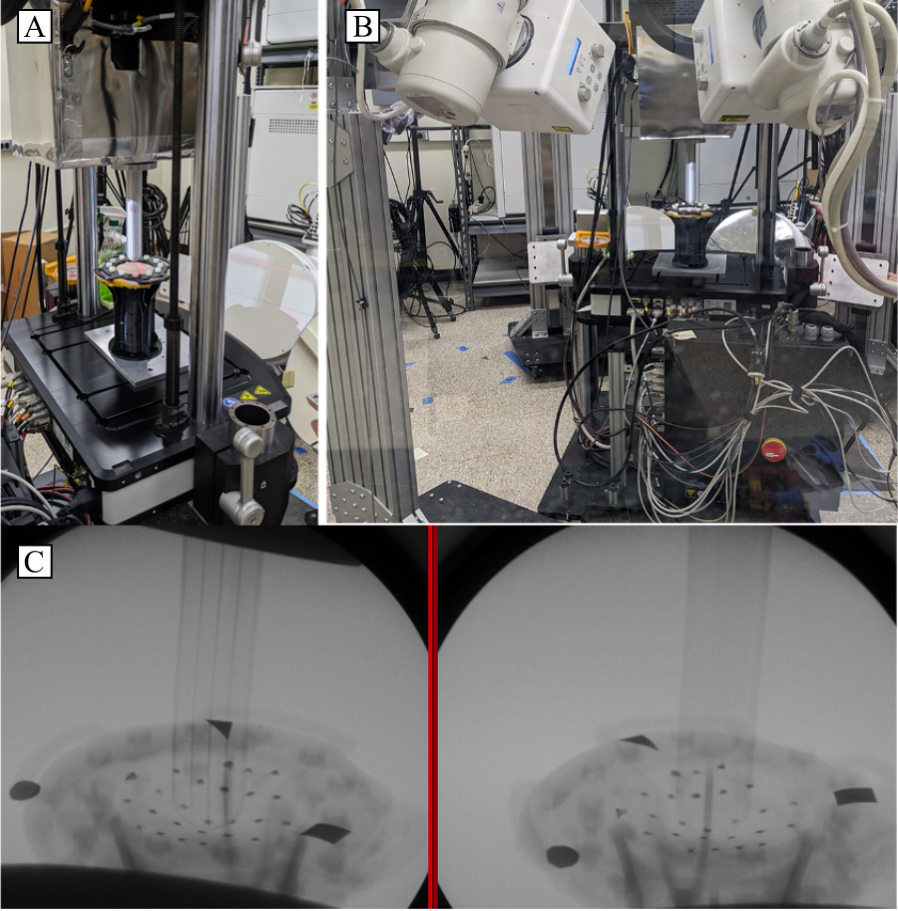
Validation apparatus setup. (A) The mechanical testing system interfaces with the dynamic stereo x-ray system. (B) Full view of the testing setup showing testing inclination. (C) Single static biplanar x-ray images of the skin deflected during compressive testing.

In conjunction with the cadaver experiments, to validate the accuracy of proposed methods to measure skin deformation, a customized, mechanical testing setup (Acumen 3AT axial-torsion system, MTS Systems Corp), which can directly interface with the DSX system, will be used. This system permits mechanical testing of soft tissue to be simultaneously performed during DSX data captures (Figure 4). This setup will be used to apply displacements to cadaveric lower limb tissue (mimicking tissue deformation in the socket) to validate the measurements from the proposed DSX analysis. To perform this validation test, 5 segments of cadaveric skin and subdermal tissue will be dissected to be mechanically tested in conjunction with DSX imaging. Each tissue sample will be affixed in tension between the opposing rings of a radiolucent plastic fixture with the skin held taut between the rings using radiolucent hardware. To validate the deformation analysis, radiopaque markers used in the cadaver experiments will be painted on the tissue. A CT scan, as previously described, of each tissue sample with applied markers will be obtained before testing to model the tissue specimen and locate the markers in a 3D space. The DSX apparatus will be angled at a maximum of 30° to distinguish the markers on the horizontal surface to avoid occlusion by the metal structures of the mechanical testing system. The mechanical testing compression rings will allow translation while the center of the fixture below the sample will be open to allow the tissue to freely deform with the applied force. Both the plastic circular fixture attachment and compressive bending fixture will be fabricated from polylactic acid thermoplastic, which is radiolucent. The Acumen 3AT axial-torsion system will be used for mechanical testing with a maximum axial testing capacity of 3 kN. To remove the effects of tissue freezing, the samples will be preconditioned at 0.1 mm/s with an axial displacement loading between 0.1 and 0.5 cm, for 50 cycles, based on the results of a previous study [32]. This displacement (0.5 cm) corresponds to the minimum bone-socket displacement associated with patellar straps, sleeves, or suspension liners [11]. Following this, the displacements associated with vacuum (1.3 cm) and traditional suction (1.8 cm) for the bone-socket interface [11] will be tested for 10 cycles each in compression at a displacement rate of 1 mm/s [32]. The axial displacement, axial force, and time will be recorded with high-speed data acquisition while the tissue is simultaneously imaged by the DSX system. A digital input-output connection will be used to synchronize the 2 systems. The compressive force on the skin tissue is proportional to the distance from the origin of the bending load, and the skin strain and shear stress can thus be calculated for a given symmetric, circular geometry. The skin strain will be compared directly to the strain calculations derived from the position of the markers collected by the DSX system. To account for the asymmetry present in the skin, the geometries, boundary conditions, material properties, and the loading regimen will be simulated using finite element analysis (Ansys, Inc). This analysis will be compared to the mechanical testing results along the skin to determine the degree to which the asymmetry of the skin affects the strain measurements.

### Clinical Experiment

Upon completion of the cadaver trials, a robust marker set will have been developed to evaluate skin strain and residual tibial movement for human participants as part of the clinical experiments. The residual tibial motion will be compared relative to the prosthetic socket for individuals with transtibial limb loss while separately using 2 suspension methods: EV suspension and traditional suction (EV system not activated). Participants will be assessed while walking at a self-selected speed on a treadmill, at a speed 10% faster, and during a step-down task. These activities were chosen for three distinct reasons: (1) their relevance to normal activities [33], (2) the fact that the forces exerted on the lower limb during stepping and walking can displace the prosthetic socket relative to the residual limb, and (3) these movements are suitable for recording by the DSX system. These 2 common suspension conditions (EV and traditional suction) were chosen because of their clinical relevance and expected measurable differences between these conditions.

### Recruitment and Enrollment

The study sample will consist of 21 individuals with unilateral transtibial limb loss recruited from Veterans Affairs New York Harbor Healthcare System. The inclusion and exclusion criteria are presented in Textbox 1.

**Inclusion and exclusion criteria.**

**Inclusion criteria**

- Veteran, service member, or civilian with unilateral transtibial limb loss of any etiology
- Aged ≥18 years
- ≥6 months after limb loss
- Current prosthesis users for ≥6 hours per day

**Exclusion criteria**

- Inability to tolerate socket suspension
- Length of the residual limb prohibits socket fitting, marker placement, or dynamic stereo x-ray data capture
- Mental impairment that impedes study compliance
- Cognitive deficits or mental health problems that would limit the ability to participate fully in the study protocol
- Skin conditions and those with severe contractures that prevent prior prosthetic wear
- Neuropathy, uncontrolled diabetes, receiving dialysis, insensate feet, severe phantom pain, a history of severe skin ulcers, or any other significant comorbidity that would interfere with the study
- Severe circulatory problems including peripheral vascular disease and pitting edema
- Women who are pregnant or who plan to become pregnant during participation in the study

### Ethical Considerations

This study was approved by the Veterans Affairs New York Harbor Healthcare System institutional review board (protocol ID 1655582) and the Rutgers University institutional review board (protocol ID Pro2022001576). All participants will provide informed consent before participating in any study activity. No research data will be acquired until the informed consent form has been signed. The principal investigator or study staff will explain the protocol, and participants will be given adequate time to review and comprehend all information before agreeing to participate in the study. After the informed consent form is signed, the study prosthetist will then screen each participant to confirm all inclusion and exclusion criteria and will inspect the residual limb to ensure that there are no issues that could prevent socket fitting. The appropriate residual limb measurements will be taken, and the residual limb will be cast to prepare for socket fabrication. Each participant’s involvement will last approximately 8 weeks, including 4 weeks for socket fabrication and fitting, a 4-week acclimation period, and a data collection visit for CT scans and DSX testing on both socket suspension conditions. Study participants will be compensated US $50 for each of the socket fitting visits (up to 4 maximum) and US $100 for the 1-day data collection and testing at Rutgers. Compensation will be given in the form of a direct deposit voucher, processed through the Veterans Affairs New York Harbor Healthcare System fiscal department.

To protect the privacy and confidentiality of the human participants, each participant will be given a unique identification number that will be used in all study-related paperwork. The specific code system will not contain any personally identifiable information. All information collected throughout the study will be methodically recorded, handled, and stored to allow for accurate reporting, interpretation, and verification. Veterans Affairs (VA) complies with the requirements of the Health Insurance Portability and Accountability Act of 1996 and its privacy regulations and all applicable laws that protect the privacy of research participants. The principal investigator and study staff will ensure that research records are stored in a manner that protects the confidentiality of human participant information. All electronic data collected will be deidentified and will not contain any Health Insurance Portability and Accountability Act identifiers, and the physical study forms (ie, signed consent forms and data collection forms) will be stored in secured and locked filing cabinets at the on-site office of the principal investigator. Computer files will be secured through password protection. Throughout the study, deidentified data (video data and electronic data) will be shared using VA information security office–approved secured VA computer systems with password protection and firewall. The study materials will be stored in accordance with the VA record control schedule.

### Prosthetic Suspensions

Individuals with transtibial limb loss will be provided with a prosthesis capable of being suspended via both EV and traditional suction, with the prosthetic ankle-foot devices they currently use. Participants will be tested under 2 conditions: with the EV suspension active and with the EV inactive (traditional suction suspension). The order of the conditions will be counterbalanced.

### Socket Fitting and Prosthetic Alignment

Before fitting the prosthetic socket, the study prosthetist will capture measurements of the residual limb (eg, length, circumference, and percentage of the sound limb). The study prosthetist will also document any areas of potential concern on the residual limb (eg, excessive redness, scar tissue, redundant tissue, heterotopic ossification, sensitivities, or evidence of previous wounds). A total of 2 to 3 preliminary “check” sockets will then be fabricated to ensure proper fit before the fabrication of the definitive socket. Each socket will be fitted with a Harmony vacuum pump (Ottobock Inc). Continued fittings will occur, as needed, to get to a comfortable, consistent daily volumetric fit throughout several consecutive days. Each participant will use their own ankle-foot device. Once a consistent, comfortable fit is achieved, fabrication of a definitive, laminated study socket will occur. Participants will use the study socket for a minimum period of 4 weeks before DSX testing occurs. Participants will be asked to use each suspension method equally during the 4-week acclimation period.

### Subjective Surveys

Following the 4-week acclimation period, participants will complete 3 surveys for each suspension method. Participants will complete the Prosthesis Evaluation Questionnaire (PEQ), which is a self-reported visual analog-style survey for individuals with lower limb loss [34]. The PEQ has 9 independent domains as well as separate, non–domain-specific questions that can be evaluated individually. For this investigation, the domains for utility, appearance, sounds, residual limb health, and ambulation will be used. The PEQ questions on satisfaction and pain will also be assessed. In addition, participants will complete the PEQ Addendum, which asks 2 open-ended questions regarding any falls and stumbles that the participant may have experienced over the previous 4 weeks [35]. To specifically assess socket comfort, participants will complete the socket comfort score scale, which is a numerical scale of socket comfort that has shown good repeatability and sensitivity to change [36].

### DSX Experimental Design

Following the 4-week acclimation period, the participants will be evaluated at the Rutgers New Jersey Medical School DSX facility. To evaluate the 6 DOF kinematics of the residual limb within the socket, participants will separately walk on a treadmill at a self-selected speed, at a speed 10% faster, and during a step-down task from an 18 cm–high platform. The DSX system will be oriented such that all tasks can be recorded with the same configuration. These movement tasks will be completed under 2 conditions: with the EV suspension active and with the EV system inactive (traditional suction). For testing with the traditional suction suspension (EV inactive), the vacuum connector will be replaced with a PushValve (Ottobock, Inc) [11]. A randomized block, crossover design will be used to evaluate the residual limb-socket fit. A group of 11 participants (group 1) and a group of 10 participants (group 2) will be formed. Participants in group 1 will first be tested in the DSX system with the EV on, and then, they will repeat all procedures with the EV inactive. Participants in group 2 will first be tested in the DSX system with the EV inactive, and then, they will repeat the tasks with the EV active. After each suspension method is applied, participants will be instructed to perform normal daily activities for 2 hours to allow for acclimation and for transient changes (eg, volume loss) to manifest. A period of 2 hours was chosen so that both conditions can be tested on the same day while still capturing 90% of the volume loss during socket use [1]. Up to 4 trials of DSX will be conducted for each task for each socket condition, giving a maximum of 24 trials per participant. For each task, DSX data will be collected and analyzed to determine the underlying bone movement and skin strain with respect to the socket. Separate CT scans of the residual limb and socket will also be captured for each participant to generate participant-specific socket and bone models of the residual limb for tracking kinematics.

DSX technology has a limited field of view (about the size of a basketball). Careful placement of the x-ray sources will permit a view of the knee, tibia, and socket. Because participants will be walking on a treadmill, the DSX setup will likely be able to capture the entire gait cycle; but at a minimum, stance phase will be collected. The 3D kinematics of the thorax, pelvis, lower limbs, and prosthesis will be recorded synchronously using a 6-camera motion capture system (Qualisys Inc). Marker-based motion capture will be used to record the overall position and orientation of the body segments that are out of the viewing volume of the DSX during the movement to establish the participants’ overall movement patterns.

### Clinical Study CT Scans

After the radiopaque markers have been applied to the residual limb and liner, the socket with embedded brass beads will be donned by the participant and a single CT scan of the residual knee joint and tibia will be acquired while participants lie in the supine position with their socket and liner on and with the knee extended. CT scans provide gray-scale volumes for use with DSX and high-resolution 3D surface models required for computing all outcome variables. CT volume images will be acquired at a resolution of 512×512×0.625 mm^3^ (120 kVp; SMART mA). Surface3D (C-Motion Inc) will be used to segment 3D models of the socket, liner, and tibia from the CT volumes.

### Quantification of the Relative Motion Between the Residual Bone and the Prosthetic Socket

Quantification of the 3D position and orientation of the residual limb has been previously described in detail [23]. In brief, the residual tibia will be tracked throughout the gait cycle, with the coordinate system defined in the CT space using a morphology-based coordinate systems adapted from previous studies [37,38]. These coordinate systems will be registered between Visual3D (C-Motion, Inc) and CT space using the coregistration transformation matrix. The position and orientation (pose) of the socket will also be tracked with 2-mm brass beads secured to the exterior of the prosthetic socket. To provide a context for the relative motion of the residual tibia and socket, 3D infrared motion capture will synchronously be recorded with the DSX data for each participant. Anatomical frames of the thorax, pelvis, and thigh will be constructed by placing retroreflective markers on anatomical locations. Visual3D will be used to establish the anatomical reference frames, estimate the position and orientation of all segments, and compute the kinematics. The reference frames for the socket will be established based on the sagittal plane of the prosthesis. The sagittal plane will be defined by the long axis of the foot. The axial direction will be defined by the pylon. The lateral direction will be perpendicular to the sagittal plane. The anterior direction will be the cross-product of the axial and lateral directions.

To use the same reference frames for both imaging techniques, the global coordinate systems of the optical motion capture and DSX systems will be coregistered by simultaneously capturing the static position of a rigid lattice consisting of 11 radiopaque, spherical markers outfitted with retroreflective tape. The transformation matrix for the coregistration of each global coordinate space will be computed and applied to the respective kinematic data sets. The pose of the socket will be estimated using a standard 6-DOF pose estimation with a set of beads as tracking markers implanted into the socket. The socket reference frame will be established by structural landmarks on the prosthesis in the CT image.

### Data Processing

**Figure 5:**
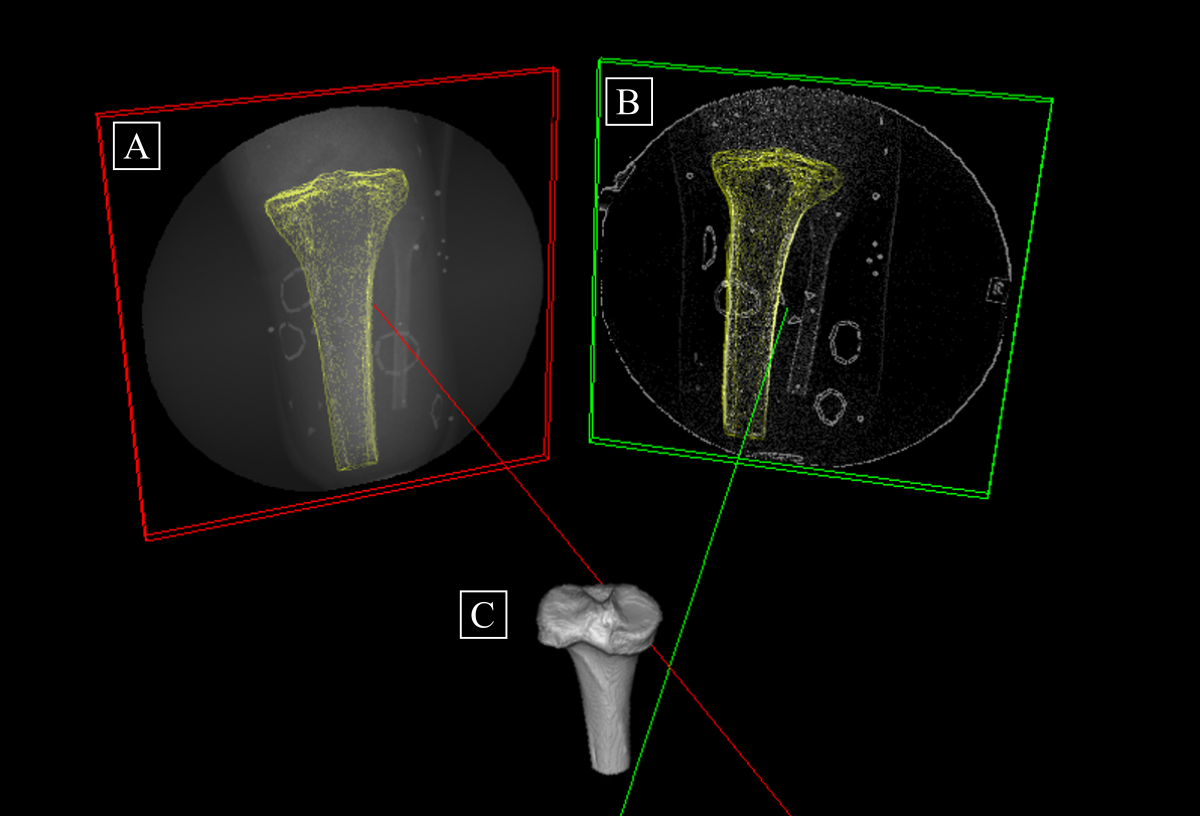
A 3D representation of the synchronized x-rays. The inline x-ray image is the red frame (A), whereas the offset x-ray image is the green frame (B). The 3D residual tibia (C) is reconstructed from computed tomography (CT) data. The red and green lines are the perpendiculars from the x-ray image planes to the x-ray sources. The outlines of the tibia (shown in yellow) superimposed over each x-ray image are created by casting rays from each x-ray source through the CT tibia to the x-ray planes.

Validation of DSX systems for 3D volumetric model-based tracking has been previously reported [39-41]. For each participant, the residual tibia will be segmented from the CT volume to construct a polygonal mesh of the tibia surface. Digitally reconstructed radiographs (DRRs) created from participant-specific CT scans will be matched to the DSX images in the inline and offset views to calculate the 3D pose of the tibia (Figure 5) [18,41]. DRRs will be generated by positioning the segmented CT volume within a virtual x-ray system and projecting rays through it to create a simulated x-ray image [23]. The optimal position and orientation of the bone will be defined as the pose that maximizes the similarity between the pair of DRR images and their corresponding x-ray images. To track each trial, the tibia will first be manually positioned in every fourth frame of data. Subsequently, a cubic spline will be fitted to the set of manual positions and orientations of the bone, which can be interpolated by the model-based tracking algorithm to determine a pose of the bone in every frame.

The algorithm output will be a set of 4×4 transformation matrices representing the pose of the residual tibia. The position and orientation of the socket will be calculated using beads placed on the rigid socket for each time frame. Excursions for relative tibial rotations and translations will be defined as the difference between the maximum and minimum values for each variable within the gait cycle. The differences between the residual bone movement with the EV and traditional suction will be determined in 6 DOF for each participant. Group differences for tibial rotations and translations will be compared at initial contact, toe-off, and swing phase as well as for the total excursions of the residual tibia during the gait cycle.

### Quantification of Skin and Liner Deformation

Motion between the bone and the skin (skin deformation) and motion between the skin and the inner surface of the liner will be examined simultaneously with bone movement in the prosthetic socket. Motion between these interfaces can be directly associated with discomfort while wearing a prosthesis [3,30]. In addition, motion between the socket and both the skin and inner surface of the liner will be examined for a complete understanding of the motion between the limb and the prosthesis.

The results from the cadaver experiments will inform the final placement of markers on the liner and skin. The final number, sizes, shapes, and placement of markers will be based on the results of the cadaver experiments and any potential overlap or bone occlusion. However, the proposed shapes will make identification of the markers in the x-ray images easier and will foster the ability to examine both shear and compression. The ends of each line will be digitized manually from the 2 sets of x-ray images. Reconstruction of the 3D trajectories of the markers will be performed using the DSX software (C-Motion Inc). Visual3D will be used to estimate the deformation of the skin and liner from the relative movement of the markers in the socket reference frame.

### Motion of the Skin and Interior of the Liner Relative to the Socket and Residual Tibia

The ends of the lines on the skin and liner will be tracked during the dynamic trials, and their trajectories in the socket coordinate system will define the motion of the skin and liner relative to the socket. Their positions in the socket coordinate system during the static trial will serve as the baseline positions. Visual3D contains the tools to define the socket coordinate system and track the motion of the skin markers within this reference frame. The motion of the lines will also be calculated in the coordinate system of the residual tibia. Their positions in the tibia coordinate system during the stationary trial will serve as their baseline positions. The tibia coordinate system will be defined to track the motion of the skin and liner markers within this reference frame.

### Deformation of the Skin

Deformation of the skin will be evaluated in the same way as in the cadaver experiments. In brief, 2 primary metrics will be used to quantify skin deformation: shear and compression. For shear, the end points from the lines will be digitized and the 3D trajectories will be used to reconstruct the 3D shape of the line at each time frame. The angles between the lines will be measured in the static pose to quantify the baseline skin shear. The change in angle will be measured during each dynamic task. Shear will be measured as the change in angle relative to the angle in the static position. Compression will be calculated by the change in distance between lines as well as a neutral position. As with shear, baseline distances will be calculated from the static trial.

### Data and Statistical Analysis

#### Overview

Descriptive statistics will be used to summarize demographic and continuous baseline variables. Median and IQR will be used to classify nonnormal or ordinal data. For the clinical experiment, a between-participant factor at 2 levels (EV on and EV off) will be used. The interaction of the suspension condition (EV on or off) will determine the efficacy of the EV system on the outcome measures.

#### Bone Movements

The differences between the residual bone movement with the EV and the traditional suction suspension will be determined in 6 DOF for each participant. Briefly, group differences for tibial rotations and translations as well as total excursions of the bone for each suspension condition will be compared during the gait cycle. Paired *t* tests will be performed and a between-participant factor at 2 levels (EV on and EV off) will be used. A hierarchical linear model will be used to evaluate the tibial range of motion (ie, maximum translation and rotation) as a function of tibial position and potential confounding factors (eg, gender, age, etiology, and time since limb loss). The hierarchical linear model will also be used to evaluate the tibial motion path and the effect of rate during the 3 different activities.

#### Skin and Liner Deformation

The characterization of 3D skin deformation will be performed by comparing the 3D position of the skin markers at rest, or unloaded position, to the 3D positions of the markers when deformed during each task to produce relative strain assessments for marked regions of the residual limb. The characterization of 3D deformation will be measured between the suspension methods. Paired *t* tests will be performed as previously described for bone movements. Linear regression models will be used to evaluate the potential confounding factors.

#### Power Analysis

Table 1 outlines the sample size estimate required to achieve 90% power for the key variables of interest. A previous pilot investigation for individuals with transfemoral limb loss [23] indicated an effect size of 0.77 for residual femur axial translation. Assuming an α of .05, using paired *t* tests, 21 participants are required to achieve 90% power to detect a 0.5 cm difference in axial translation. Owing to the paucity of highly accurate data on movement between the residual tibia and socket, data from Board et al [1] were used to calculate an effect size for tibia translation (1.75) for suction and EV suspension. Given an effect size of 1.75 and an α of .05 for a paired *t* test, a sample size of 21 will be powered at >99% to detect differences between EV on and off. For skin strain, Gale et al [30] found a maximum shear strain of approximately 0.08. Assuming a shear strain of 0.08 with a moderate effect size of 0.75 (α=.05), a sample size of 21 will be required to achieve 90% power to detect differences between EV on and off.

**Table 1:** Power analysis.

Measure	Values, mean (SD)	Sample size for achieving 90% power, n
Femur distal translation (cm)	2.0 (0.6)	21
Tibial translation (cm)	4.0 (0.4)	6
Skin shear strain	0.08 (0.02)	21

## Results

This study was funded in October 2021. Cadaver testing began in January 2023. Enrollment began in February 2024. Clinical data collection is expected to be completed in December 2025, and the study is expected to be completed in April 2026. Data analysis of the full data set will begin after final data collection. Initial dissemination of results is expected in November 2026, with subsequent publication of secondary analyses in 2027.

## Discussion

### Expected Outcomes and Anticipated Principal Findings

It is expected that the outcomes of this investigation will significantly contribute to the understanding of the complex mechanics of the residual limb-tissue-socket interfaces during dynamic activities for individuals with lower limb loss. It is also expected that by using DSX in combination with novel mathematical algorithms, relative movement between the residual limb and socket and skin deformation can be accurately measured during dynamic motions for individuals with lower limb loss. Ultimately, this foundational information can be critical for developing a database of biomechanical socket parameters deemed important for socket fit, limb health, and comfort. By using the techniques developed in this investigation to perform future comparative effectiveness research of current prosthetic socket technology, better information about the benefits, risks, and costs of different socket options can be generated to provide health care decision makers (eg, patients, clinicians, and policy makers) with highly accurate, evidence-based information. Furthermore, the methodology detailed in this study should speed up the processing time, which, to date, has been burdensome for researchers. This will allow for faster dissemination of information and greater throughput of data to inform clinical guidelines for prosthetic suspension systems. Finally, this investigation can provide vital foundational information that can be used by leading manufacturers in prosthetic design to create enhanced socket technology that has the potential to reduce long-term secondary physical comorbidities and degenerative changes [42].

As the number of individuals with limb loss continues to grow [43], substantial resources will be required for rehabilitation and prosthetic services for this population. Effective outcomes-based clinical practice will be necessary to reduce long-term disabilities associated with prosthetic use. As such, it is the objective of this investigation to examine the dynamic in vivo kinematics between the residual limb and prosthetic socket in 6 DOF of motion, as well as to quantify residual tissue and liner deformation for individuals with transtibial limb loss.

### Dissemination Plan

For large-scale dissemination in the Department of Veterans Affairs and Department of Defense, results will be presented through webinars offered by the Extremity Trauma and Amputation Center of Excellence, which are available across the entire Department of Veterans Affairs and Department of Defense health care networks. These webinars are offered to researchers and health care professionals who provide care for individuals with limb loss. For stakeholders in the civilian health care systems, the outcomes and important techniques developed in this study will be published in highly rated peer-reviewed scientific journals (eg, *Gait & Posture, Clinical Biomechanics, Journal of Biomechanics, Frontiers,* and *Orthotics and Prosthetics International*). The results will also be presented at professional conferences that are specific to clinical limb loss care teams (eg, the American Academy of Orthotists and Prosthetists, American Society of Biomechanics, and Military Health System Research Symposium). The findings and methods of this study will also be directly distributed to industry partners. Because most prosthetic technologies used by veterans and service members with limb loss are developed by industrial entities, sharing new evidence-based information with industry leaders will support the development of future products to better meet the needs of individuals with lower limb loss.

### Limitations

The heterogeneity of the population, including varied ages, etiology of limb loss, prosthesis experience, and time since limb loss, may limit the generalizability of the outcomes to a more diverse population. However, the statistical analysis models will adjust for these specific confounding factors. Although participants will use the same type of prosthetic socket, the ankle-foot devices will not be prescribed, which could introduce additional variability. The tibia and skin markers will likely be manually positioned at every fourth to tenth time frame, which may cause intratracker errors that could be improved with automated algorithms. Finally, the limited field of view for DSX may restrict collecting data for the entire gait cycle and will be unable to record the overall position and orientation of the lower limbs and pelvis that are out of view.

## Appendix

Multimedia Appendix 1 Peer-reviewed summary statement from the Congressionally Directed Medical Research Programs, Orthotics and Prosthetics Outcomes Research Program.

## Abbreviations

CT: computed tomography
DIC: digital image correlation
DOF: degrees of freedom
DRR: digitally reconstructed radiograph
DSX: dynamic stereo x-ray
EV: elevated vacuum
PEQ: Prosthesis Evaluation Questionnaire
VA: Veterans Affairs

## Data Availability

All data generated or analyzed during this study are included in this published paper. The final results will be available via a publicly available data repository.
