# Supplementary material for: Quantifying Bone and Skin Movement in the Residual Limb-Socket Interface of Individuals With Transtibial Limb Loss Using Dynamic Stereo X-Ray: Protocol for a Lower Limb Loss Cadaver and Clinical Study
Source: JMIR Res Protoc. 2024 Apr 26;13:e57329. doi: 10.2196/57329 (PMC11087852; doi:10.2196/57329)
Supplement: Multimedia Appendix 1 [file resprot_v13i1e57329_app1.pdf]

SUMMARY OF DISCUSSION

A Subcommittee of the Rehabilitation Research and Development Service Scientific Merit Review Board met in Plenary Session and reviewed the above proposal considering all internal and external reviews. This document summarizes the major points of the discussion concerning the proposed project. In any further development of this project, the investigator should consider carefully all the issues reflected in this Summary of Discussion as well as the more detailed comments in the individual critiques.

GENERAL COMMENTS:

The goal of the proposed investigation is to study the dynamic, *in vivo* kinematics between the residual limb and the prosthetic socket and also to quantify the 3D deformations in the skin and liner in individuals with trans-tibial amputations. Excessive movement of the limb and skin/liner deformations can lead to pain, gait deviations and discomfort. Assessing this *in vivo* relative motion in dynamic activities will be useful for designing the right socket for the right patient. If successful, the technology has the potential to reduce the complications and treatment costs associated with excessive limb/socket motion and lead to significant improvement in the quality of life of people living with lower limb loss.

The authors have addressed concerns brought up by previous reviewers. The inclusion of additional cadaveric testing to validate the relationship between estimated shear with the DSX system and measured in mechanical testing will add validity to this methodogical system, and the increase in sample size will ensure statistical power to address Aims 2&3. The reviewers appreciate the rationale for not including a third suspension condition.

SUGGESTIONS:

Although the technology proposed is impressive and the challenge being addressed important, the translation of this technology and its potential to directly impact care in a meaningful way, remains the primary concern. Although there is little doubt this work will be able to produce fundamental knowledge on limb/socket issues, how might this knowledge be used is less defined. Certainly, the ability of using this technology to perform comparative effectiveness studies will help with decision making and the ability to use this technology to calibrate FEA models will also help with defining limb/socket interactions and potentially drive better socket designs. Yet, what will this research group do to drive that forward? What are the next steps for these researchers to drive translation? There was no clear presentation of what this group of investigators would do next if this project was successful.

Although the applicants describe how patient’s limbs will be monitored for skin irritation, the patient studies could be strengthened by more formally and rigorously evaluating for signs of skin irritation and patient discomfort by comparing differences in residual limb complications/bone positioning with the DSX results. This analysis would raise confidence that the analytical tool proposed, may also be sensitive enough to confirm any differences in the problematic clinical tissue responses highlighted in the introduction. A more robust experimental study question that investigates DSX, skin/bone movement and any patient discomfort (and potentially its relationship to some long-term clinical outcome) would strengthen the proposal by supporting the potential role of DSX in patient care.

Limb volume, shape of the residual limb, tissue composition, and the time of day are variables that will influence the interaction between the socket and the limb. These variables should be accounted for in the design of the study and interpretation of results.

Finally, the low publication rate of the team is a concern and raises questions about the outcomes of the study, if funded.

COMMENTS ON THE BUDGET:
None.

DESCRIPTION (provided by applicant):
Individuals with lower extremity amputation (LEA) often experience relative motion between their residual limb and the prosthetic socket, such as vertical translation and axial rotation, which can cause inefficient dynamic load transmission from the distal prosthetic components to the residual limb. This can lead to significant secondary consequences, such as pain, gait deviations, and discomfort that limit mobility and autonomy. Assessments of the relative motion between the bone and the prosthetic socket have been performed, but there is little existing data on dynamic, *in vivo* residual limb-socket kinematics since most investigations have been performed using non-dynamic testing protocols, static measurements, or with unvalidated surface marker-based motion capture systems. Dynamic Stereo X-ray (DSX) is an advanced imaging technology that can quantify 3D bone movement and tissue/liner deformation inside a prosthetic socket during dynamic activities. It can achieve sub-millimeter accuracy of bone pose (position and orientation) measurement during functional movements by combining 3D models derived from CT scans with movement data from biplanar x-ray video. There is a substantial gap in our understanding of the complex mechanics of the residual limb-socket interface during dynamic activities that limit the ability to improve prosthetic design. Our 4-year goals for this project are to develop the analytical tools to quantify both the dynamic, *in-vivo* kinematics between the residual limb and socket, as well as the mechanism of residual tissue/liner deformation. In order to validate the sensitivity of this methodology to differences in socket suspension, we will evaluate 2 suspension systems: elevated vacuum (EV) and simple suction. We hypothesize that an efficient and highly accurate method to quantify the dynamic interaction between the residual limb and prosthetic socket will be sensitive enough to distinguish between different types of prosthetic socket suspension, which will further the biomechanical understanding of socket design. To do so, the investigators will address the following aims: (1) To optimize the DSX procedural setup for the accurate tracking of the prosthetic socket, skeletal kinematics, and tissue/liner deformation; (2) To quantify the relative motion between the residual tibia and the prosthetic socket during dynamic activities; and (3) To measure the deformation of the skin and liner in the prosthetic socket during dynamic activities. To address these aims, we will first employ a cadaver study to optimize the placement of an array of radio- opaque beads and markers on the socket, liner, and skin to simultaneously assess both dynamic skeletal movement and residual tissue/liner deformation. Five cadaver limbs will be utilized in an iterative process to develop an optimal marker setup. Stance phase gait will be simulated during each DSX session to induce bone movement and skin/liner deformation. The number and placement of markers will be evaluated after each session to refine the marker placement to track skin/liner deformation and skeletal movement. Once an optimal marker setup is identified, 21 subjects with transtibial amputation will be fit with a socket capable of being suspended via both EV and traditional suction. Subjects will undergo a 4-week acclimation period and then be tested at the DSX facility at Rutgers University. DSX will be utilized to track skeletal and skin/liner motion under both suspension techniques during 3 dynamic activities: treadmill walking at self-selected speed, fast walking (10% faster), and a step-down movement. The performance of the two suspension techniques (active EV and normal suction) will be tested by quantifying the 3D bone movement of the residual tibia with respect to the prosthetic socket and quantifying liner and soft tissue deformation at the socket-residuum interface. By using the analytical tools for a highly accurate, *in-vivo* assessment of residual limb-socket motion, we can provide vital foundational information to aid in the development of new methods and techniques to enhance prosthetic fit that have the potential to reduce secondary physical comorbidities and degenerative changes that result from complications of poor prosthetic load transmission.

PUBLIC HEALTH RELEVANCE:

Individuals with lower extremity amputation are often challenged by complications that arise from poor prosthetic fit, including movement of the residual limb in the socket, known as pistoning. Pistoning can lead to gait instability, skin problems, and pain. Different prosthetic suspension systems have been developed to decrease this motion, including elevated vacuum suspension, which utilizes a pump to draw air from the socket. However, scientific analyses to understand the movement between the limb and socket in elevated vacuum systems have yet to be performed with a high level of accuracy. This study will use a state-of-the art imaging technique, known as dynamic stereo x-ray, to quantify the 3D movement of the residual limb in the socket. This information is critical for advancing prosthetic treatments to reduce secondary conditions and degenerative changes that result from poor prosthetic fit.

CRITIQUE 1

Significance and Innovation: This proposal seeks to examine the dynamic, *in-vivo* kinematics between the residual limb and prosthetic socket, as well as quantify deformation of the skin and liner for individuals with transtibial amputation (TTA). This work is significant in that the dynamics at the limb/socket interface are poorly understood yet the stresses and strains imposed on the tissues within the residual limb may be the underlining cause to multiple socket fit and tissue health issues experienced by prosthetic socket users. The work here is fundamental to understanding those relationships, it builds from previous pilot work in people with transfemoral amputation and is tackling a very challenging technical problem. The methodology presented here innovates from this previous work in a way that offers a more robust, and logical approach to its development and the inclusion of different tasks and suspension systems to better define the range of which this methodology could be applied while being clinically relevant. The authors have made many positive changes since the last review and addressed multiple concerns brought up by the reviewers, yet the primary way significance could be approved remains on the clinical translational side.

Importance and Impact: Challenges with socket fit and maintaining tissue health inside of a prosthetic socket that is subjecting the skin and underlining tissues to loads that tissue was never intended to handle creates a slew of limb health issues that continually plague those that use a prosthesis. At the end of the day, it is rarely the foot or some other widget placed under the socket that prevents someone from the using their prosthesis to participate in their community, it is the residual limb tissue itself and its propensity for breakdown. This was not only highlighted by the references in the background of this application but is certainly in line with the clinical experiences of this reviewer. Overcoming these challenges will require a detailed and fundamental understanding of tissue dynamics at the limb/liner, and liner/socket interface. This knowledge will, in turn, enable a better characterization of tissue strains in the residuum and thus provide the fundamental knowledge that will lead to a variety of potential solutions. One example could be the identification of how the mechanisms of skin breakdown at the residual limb. Fundamental study of limb/socket tissue dynamics should provide a way to focus future efforts on this topic.

Contribution to VHA: The methodology developed here will have a broader, more indirect, and longer time frame until that impact is truly achieved within the VA. When viewed in the context of the challenges facing optimization of the limb/socket interface and the impact of maximizing tissue health at this interface and its potential impact to improve function and quality of life for Veterans living with amputation, this research has enormous positive potential to contribute to the VA.

Methods (Data Analysis)**:** The authors have addressed concerns brought up by previous reviewers. The inclusion of additional cadaveric testing to validate the relationship between estimated shear with

the DSX system and measured in mechanical testing will add validity to this methodological system, and the increase in sample size will ensure statistical power to address Aims 2&3. I appreciate the thought and rationale to not including a third suspension condition.

Adequacy of Data: The study seems well poised for success.

Project Organization and Management: The authors adequately addressed my concerns on participant transportation.

Investigator Qualifications: The investigators appear well qualified to handle this project.

Facilities and Resources: The facilities appear adequate.

Human Subjects: The human subject plan appears adequate, the exclusion of women that are or plan to become pregnant is warranted given the change in gait and risk to the fetus.

Critique of Vertebrate Animals Section:

	Yes	No
Research with vertebrate animals?		X

Biohazards and Radioisotopes: The proposal includes steps to minimize radiation exposure and appropriate measures to maximize safety for the participants and staff.

Inclusion of Women, Minorities and Children: This study will exclude women that are or planning to become pregnant due to safety concerns for the fetus and radiation exposure. This exclusion is warranted.

Budget (unscored)**:** The level of commitment and personnel costs seem appropriate for a project of this size.

Data Management and Access Plan (for data sharing, unscored)**:** Data management plan appears sufficient.

Overall Strengths: This work is significant in that the dynamics at the limb/socket interface are poorly understood yet the stresses and strains imposed on the tissues within the residual limb may be the underlining cause to multiple socket fit and tissue health issues experienced by prosthetic socket users. The work here is fundamental to understanding those relationships, it builds from previous pilot work in people with transfemoral amputation and is tacking a very challenging technical problem.

Overall Weaknesses: Indirect clinical translation remains the primary weakness. Although there is little doubt this work will be able to produce fundamental knowledge on limb/socket issues, how might this knowledge be used is less defined (although better defined than in previous submissions). Certainly, the ability of using this technology to perform comparative effectiveness studies will help with decision making and the ability to use this technology to calibrate FEA models will also help with defining limb/socket interactions and potentially drive better socket designs. Yet, what will THIS research group do to drive that forward? What are the next steps for these researchers to drive translation? This development is a long term investment, and this research group is best positioned to leverage this

knowledge for the next steps, and yet, I was unable to find a clear vision on what this group would do next when this project was successful.

CRITIQUE 2

Significance and Innovation: This proposal aims to use dynamic stereo X-ray (DSX) to generate analytical data that will accurately measure relative dynamic motion between the residual limb and socket. Movement can lead to secondary consequences such as pain, gait deviations and discomfort. The proposed technology will be developed using a cadaver study, where results will feed into a clinical study to assess *in-vivo* skin biomechanics in 6 degrees of freedom. In the clinical study, two models will be investigated; an elevated vacuum system and traditional suction suspension system as these are two methods that are commonly used in the clinic. The applicants hypothesize that the technology proposed will be sensitive enough to be able to distinguish between the two techniques.

The applicants have addressed each of the Subcommittee's previous comments and concerns in point-by-point form. The clinical implications are clearer and the future use of FEA in order to personalize care, is encouraging.

Importance and Impact: Methods that improve socket fit and reduce tissue damage to the residual limb are warranted. Presently, there is no overall satisfactory socket and novel approaches that seek to improve our understanding of the interfacial conditions as well as residual bone movement, are of high importance.

Contribution to VHA: If successful, the scientific knowledge gained may directly aid VHA amputees.

Methods (Data Analysis)**:** Concerns were previously raised regarding the extent and impact that this technology may ultimately offer patients. The applicants describe three main avenues through which their tool may impact clinical care in the future. Although the technology proposed is impressive and the challenge being addressed important, the translation of this technology and its potential to directly impact care in a meaningful way, remains a concern. Although the applicants describe how patient's limbs will be monitored for skin irritation, the patient studies could be strengthened by more formally and rigorously evaluating for signs of skin irritation and patient discomfort by comparing differences in residual limb complications/bone positioning with the DSX results. This analysis would raise confidence that the analytical tool proposed, may also be sensitive enough to confirm any differences in the problematic clinical tissue responses highlighted in the introduction.

Adequacy of Data: Appropriate and satisfactory.

Project Organization and Management: Appropriate and satisfactory.

Investigator Qualifications: The team are strong with an impressive funding track-record and experience in this area.

Facilities and Resources: The facilities and resources are suitable to ensure successful completion of the work.

Human Subjects: A thorough description of the inclusion/exclusion criteria, risks and prevention of risks has been included.

Critique of Vertebrate Animals Section:

	Yes	No
Research with vertebrate animals?		x

Biohazards and Radioisotopes: This proposal will use dual stereo X-ray techniques and CT imaging and the risk of radiation exposure (as well as prevention of risk) has be satisfactorily addressed.

Inclusion of Women, Minorities and Children: The proposal describes methods to ensure the inclusion of women and minorities in this study. The study does not include children.

Budget (unscored)**:** The budget is appropriate.

Data Management and Access Plan (for data sharing, unscored)**:** Appropriate.

Overall Strengths:
- The team is strong with significant previous clinical research experience.
- The investigators seek to improve clinical and basic science knowledge in an area of patient need.
- The application is well written and the methods and goals are clear.

Overall Weaknesses: A more robust experimental study question that investigates DSX, skin/bone movement and any patient discomfort would strengthen the proposal by supporting the potential role of DSX in patient care.

CRITIQUE 3

Significance and Innovation:
Strengths: The goal of the proposed investigation is to study the dynamic, *in vivo* kinematics between the residual limb and the prosthetic socket and also to quantify the 3D deformations in the skin and liner in individuals with trans-tibial amputations. Excessive movement of the limb and skin/liner deformations can lead to pain, gait deviations, and discomfort. Assessing this *in vivo* relative motion in dynamic activities will be useful for designing the right socket for the right patient.

Importance and Impact:
Strengths: If successful, the technology has the potential to reduce the complications and treatment costs associated with failures of socket suspension and lead to significant improvement in the quality of life of amputee patient population.

Weaknesses:
- The investigators did not make a convincing case for the clinical applicability of the technology in the short- or the mid-term. As authors acknowledge, it may be unrealistic to expect the DSX technology to be available at the clinic level in the near future.
- One of the previous reviewers brought up an excellent point that the real value of the technology being proposed maybe to provide insight into mechanisms of tissue breakdown at the skin-socket interface. This point is missed by the investigators in their revised application.

Contribution to VHA: The area of proposed research is relevant to improving the quality of life of Veterans with limb amputations.

Methods (Data Analysis)**:**
Strengths: The methodology is technically feasible.

Adequacy of Data:
Weaknesses:
- How will the various outcomes be combined to recommend which socket design is better?
- The outcomes and recommendation are likely to be dependent on subject-specific factors. How is this taken into account?

Project Organization and Management: No comment.

Investigator Qualifications: The Principal Investigator (PI) has several years of experience working in the field of lower limb prosthetics and has been consistently funded in the area.

Weaknesses:
- Unfortunately, even in the twice-revised application the Biographical Sketch of the PI is poorly organized when it comes to peer-reviewed publications. The publication list continues to include conference presentations; many of the publications are incompletely cited (without journal names) or are under review. The PI has numerous on-going or completed funded projects but the Biographical Sketch as submitted does not do justice to his experience.
- The complete lack of first-author peer-reviewed publications is very concerning. It does not bode well for a productive, peer-reviewed outcome of the application, if funded.

Facilities and Resources: There are no concerns.

Human Subjects: Human subjects will be recruited and tested for the study. The radiation exposure (arising from a CT scan and up to 24 trials of x-ray videography) seems excessive, although the investigators indicate this represents minimal risk.

Critique of Vertebrate Animals Section: No vertebrate animals are involved.

	Yes	No
Research with vertebrate animals?		X

Biohazards and Radioisotopes: No comment.

Inclusion of Women, Minorities and Children: No comment.

Budget (unscored)**:** No comment.

Data Management and Access Plan (for data sharing, unscored)**:** No comment.

Overall Strengths:
- The proposed work on assessing the prosthesis-limb interface is of importance to the patient-care mission of the VA.
- If successful, the technique or the data on different socket performance has the potential to reduce the complications and treatment costs associated with failures of socket suspension and lead to significant improvement in the quality of life of lower-extremity amputee population.

Overall Weaknesses:

- The investigators did not make a convincing case for the clinical applicability of the technology in the short- or the mid-term. As authors acknowledge, it may be unrealistic to expect the DSX technology to be available at the clinic level in the near future.
- In that context, one of the previous reviewers brought up an excellent point that the real value of the technology being proposed maybe to provide insight into mechanisms of tissue breakdown at the skin-socket interface. This point is missed by the investigators in their revised application.
- The outcomes and recommendations are likely to be dependent on subject-specific factors. How will this be taken into account?
- The PI has numerous on-going or completed funded projects but the Biographical Sketch as submitted reflects very poorly his experience. The complete lack of first-author, peer-reviewed publications is very concerning. It does not bode well for a productive, peer-reviewed outcome of the application, if funded.
